# Primary Signet Ring Cell/Histiocytoid Carcinoma of the Eyelid: Clinicopathologic Analysis with Evaluation of the E-Cadherin/*β*-Catenin Complex and Associated Genetic Alterations

**DOI:** 10.1155/2021/6628150

**Published:** 2021-11-11

**Authors:** Maria Del Valle Estopinal, Lavinia P. Middleton, Bita Esmaeli, Keyur P. Patel, Sara Nowroozizadeh, Michelle D. Williams

**Affiliations:** ^1^Department of Pathology, The University of Texas MD Anderson Cancer Center, 1515 Holcombe Blvd., Unit 85, Houston, TX 77030, USA; ^2^Departments of Pathology and Ophthalmology, Ophthalmic Pathology Division, University of California Irvine, 101 The City Drive, South Orange, CA 92868, USA; ^3^Ophthalmic Plastic and Orbital Surgery Fellowship Program, Department of Plastic Surgery, The University of Texas MD Anderson Cancer Center, 1515 Holcombe Blvd., Suite 1488, Houston, TX 77030, USA; ^4^Molecular Diagnostics Laboratory (Hematologic Malignancies), The University of Texas MD Anderson Cancer Center, Pathology and Laboratory Medicine/Hematopathology, 6565 MD Anderson Blvd., Unit 1062 Houston, TX 77030, USA

## Abstract

Signet Ring Cell (SRC)/Histiocytoid carcinoma of the eyelid is a rare neoplasm that shares histological and immunohistochemical similarities with diffuse gastric cancer and breast lobular carcinoma. The *CDH1* gene, which encodes the E-cadherin protein, is the best known gene associated with these tumors. The structural and functional integrity of E-cadherin is regulated by interconnecting molecular pathways which might participate in the development of this disease. Hence, we analyzed the protein expression in key genes in E-cadherin-related pathways associated with primary SRC/Histiocytoid carcinoma of the eyelid. SRC/Histiocytoid carcinoma diagnosed in the eyelid/orbit at MD Anderson Cancer Center from 1990 to 2016 were evaluated. Clinicopathologic findings were studied to confirm the primary site of origin. Immunohistochemical studies for the expression of E-cadherin, *β*-catenin, c-Myc, Cyclin D1, Src, and p53 were analyzed. Next generation sequencing for the detection of somatic mutations was performed on each tumor with matched normal tissue, examining 50 cancer-related genes. Four primary SRC/Histiocytoid carcinomas of the eyelid were diagnosed in four male patients aged 40-82 years. Immunohistochemically, two tumors with loss of E-cadherin expression had weak *β*-catenin and low cytoplasmic staining for Src while the other two cases with intact E-cadherin showed strong *β*-catenin expression and high cytoplasmic expression for Src. Cyclin D1 was focally positive in three cases. Somatic mutations in *CDH1*, *PIK3CA*, and *TP53* genes were detected in two cases. Our results suggest an abnormality in the convergence of E-cadherin/*β*-catenin pathways which may promote tumorigenesis by inducing expression of oncogenes such as *Cyclin D1* and *C-Myc*. Mutations in *CDH1*, *PIK3CA*, and *TP53* genes could induce E-cadherin dysfunction which takes part in the development and progression of this malignancy.

## 1. Introduction

Primary Signet Ring Cell (SRC)/Histiocytoid carcinoma of the eyelid is a rare, aggressive neoplasm favored to arise from the eccrine sweat glands. To date, less than 40 cases of SRC/Histiocytoid carcinoma arising from the eyelids have been described in English literature [1–3]. Clinically, this tumor presents as a vague painless, nodular, indurated mass with diffuse, poorly defined margins, arising most commonly in elderly men [1]. The clinical course ranges between 5 and 10 years with one or more recurrences after excision and tendency for orbital invasion [4]. Despite its protracted clinical course, the tumor is capable of producing regional and distant metastases on long-term follow-up, including involvement of the lymph nodes, skin, parotid gland, lungs, and liver [5]. Local excision, possibly necessitating radical surgery, is probably the preferred method of treatment, but local radiotherapy and chemotherapy may have a beneficial effect in retarding the spread of the disease [5]. The diagnosis can be challenging based on the nondescript clinical and imaging findings. The latter mainly demonstrates a diffuse enhancing and infiltrative mass involving eyelid structures and orbit by computer tomography (CT) scanning and/or magnetic resonance imaging (MRI). [6–10]

Histologically, this tumor shows diffuse involvement of the eyelid by predominantly single cells with a spectrum of histologic features encompassing atypical cells with intracytoplasmic vacuoles and eccentric nuclei, which exhibit signet ring cell appearance and/or cells with amphophilic cytoplasm and large nuclei resembling histiocytes. These histologic findings in the eyelid are comparable to signet ring cell carcinoma (SRCC) of the gastrointestinal tract and breast. In SRCC of gastric origin and breast lobular carcinoma, E-cadherin plays a central role in the pathogenesis, and hereditary loss of *CDH1*, a tumor suppressor gene encoding E-cadherin protein, is one of the known pathogenic mechanisms for this disease which correlates well with loss of E-cadherin expression by immunohistochemical evaluation [11]. Physiologically, E-cadherin/*β*-catenin complex is required to maintain the integrity of epithelial cell-cell contact, keeps Wnt/*β*-catenin signals in check, and is involved actively in epithelial-mesenchymal transition (EMT) and mesenchymal-to-epithelial transition (MET) which plays an important role in embryo development, tissue fibrosis, and cancer progression [12]. Loss of E-cadherin expression or loss of its normal localization at cell-cell contacts is consistently observed at sites of EMT during tumor progression and as initial step of metastasis. The E-cadherin gene can be genetically inactivated by a number of mechanisms encompassing loss of heterozygosity (LOH) on chromosome 16; E-cadherin promoter methylation; transcriptional repression binding of the *CDH1*-E box elements by the transcription factors Snail and Sip-1; and phosphorylation by tyrosine kinase such as EGFR, c-Met, FGFR, and c-Src [13–16]. The latter belongs to the nonreceptor tyrosine kinases which are crucial during tumor metastasis. In addition, abnormal expression of *β*-catenin promotes tumorigenesis by upregulating the expression of oncogenes such as *c-Myc* and *Cyclin D1* [17]. As mechanisms contributing to tumorigenesis of SRC/Histiocytoid carcinoma of the eyelid remain unknown, we analyze molecular mechanisms associated with the E-cadherin/*β*-catenin complex.

## 2. Materials and Methods

**Figure 1:**
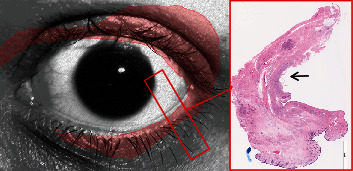
Case 3. Orbital exenteration. Circumferential sectioning of the eyelids and soft tissue allowed for mapping tumor extension which is nearly 360° (tumor spread = red area). Grossly, only slight firmness was present in the temporal/lacrimal gland area. Histopathologically, the tumor involves the dermis, palpebral conjunctiva, and periorbital soft tissue (arrow).

We searched the pathology files at The University of Texas MD Anderson Cancer Center (MDACC) from 1990 to 2016 for carcinomas of the eyelid or orbit with diagnosis of/or description that included histiocytoid or signet ring cell features. Five cases of SRC/Histiocytoid carcinoma of the eyelid and orbit were identified at our facility. Clinical and histopathologic findings were evaluated including imaging workup to exclude metastasis to this site. Metastatic adenocarcinoma of the breast to the eyelid was found in one of the 5 cases and was excluded from the analysis. The histopathologic slides of the primary four cases with SRC/Histiocytoid carcinoma of the eyelid were retrieved and analyzed. Clinical and surgical records were evaluated for reporting in conjunction with the histopathologic and immunohistochemical findings. The study was approved by the institutional review board of MDACC. The approach to the pathologic evaluation whether during immediate intraoperative assessment or for permanent processing must account for the ill-defined nature of the disease and potential widespread infiltrative single-cell pattern of growth. As performed in two cases of orbital exenteration, circumferential mapping of the eyelids around the globe at each clock designation aided in determining the extension of the disease which was beyond the clinically apparent tumor in all the cases (Figure 1).

Formalin-fixed, paraffin-embedded 4-micra unstained tissue sections were immunohistochemically analyzed with monoclonal antibodies: cytokeratin 7 (CK7) (1 : 100 Dako, Carpinteria, CA), cytokeratin 20 (CK20) (1 : 400, Dako, Carpinteria, CA), E-cadherin (1 : 1000, Invitrogen, Camarillo, CA), *β*-catenin (1 : 1500, BD Biosciences, San Jose, CA), c-Myc (predilute; Ventana Medical Systems, Tucson, AZ), Cyclin-D1 (1 : 40, Lab Vision Corporation, Fremont, CA), Src (1 : 800, from Cell Signaling Technology, Danvers, MA), p63 (1 : 1000, from Santa Cruz Biotechnology, Dallas, TX), Her-2/neu protein (RTU, clone 4B5, from Ventana Medical Systems, Tucson, AZ), and p53 (1 : 100, from Dako, Carpinteria, CA). Immunohistochemical studies were performed on an automated stainer (Leica Biosystems, Inc., BOND-III System, Buffalo Grove, IL). All antibodies and testing were performed in a CLIA-certified laboratory with standardized clinical methodology and positive control tissue.

Evaluation of each immunohistochemical marker included the cellular distribution and percentage of tumor staining. Cytoplasmic immunoreactivity was evaluated for CK7 and CK20 while nuclear immunoreactivity for c-Myc, Cyclin-D1, p63, and p53 were assessed. We defined negative as no reactivity or less than 1% immunoreactive tumor cells, focal (+) when positivity was seen in 1% to less than 25%, moderate (++) when reactivity was seen in 25% to 50%, and strong diffuse (+++) when immunoreactivity was present in more than 50% of the tumor cells, based on immunohistochemical findings of previous studies [2]. E-cadherin and *β*-catenin patterns of expression were analyzed using the adjacent normal epithelium as an internal positive control. Cytoplasmic and membranous immunostaining for E-cadherin and the pattern of membranous staining for *β*-catenin immunoreactivity were analyzed based on prior study by Karayiannakis et al.[18]. When 90% or more of the tumor cells exhibited the same homogeneous intensity as the adjacent normal epithelium, the antigen expression was considered as normal or preserved. Heterogeneous, incomplete cytoplasmic/membranous staining in more than 10% to less than 90% of tumor cells was considered abnormal expression. Altered cellular distribution of the immunostaining for *β*-catenin (cytoplasmic or nuclear) was also considered within the category of abnormal expression. Sections with 10% or less stained tumor cells or with complete absence of staining for E-cadherin and/or *β*-catenin were considered negative. Src cytoplasmic and membranous staining was evaluated using the adjacent normal keratinocytes. We defined negative as no cytoplasmic immunoreactivity, weakly positive when decreased/low cellular intensity was seen in less than 25% of tumor cells, and positive/high immunoreactivity when 25%-75% of tumor cells exhibited cytoplasmic staining with the same or higher intensity as normal adjacent epidermal cells. [19]

The interpretation of Her-2/neu status by immunohistochemistry as well as fluorescence in situ hybridization (FISH) was based upon American Society of Clinical Oncology/College of American Pathologist's criteria (2013 updated guidelines) for Her-2/Neu testing in breast cancer.

### 2.1. Molecular Studies

Molecular profiling using next generation sequencing (NGS) on the Ion Torrent PGM (Life Technologies) was performed on each tumor with matched normal tissue. Fifty cancer-related genes were examined utilizing the AmpliSeq Cancer HotSpot v2 Panel (Table 1). NGS can be performed from DNA extracted from formalin-fixed paraffin-embedded tissue (FFPE) as well as from core biopsies and fine needle aspiration material (FNA) [20, 21]. NGS allows for detection of substitutions, small insertions, and deletions.

Serial sections of formalin-fixed paraffin-embedded tissues were reviewed by our pathologists to circle the tumor-rich area and estimate tumor purity of more than 60%. DNA was extracted from a random number of unstained sections, each 0.4 *μ*m thick as described previously [21]. Deparaffinization was performed after manual microdissection of the tumor-rich area. The PicoPure DNA Extraction Kit (Arcturus, Mountain View, CA) was used to extract DNA, which was then purified using the Agencourt AMPure XP kit (Agencourt Biosciences Corporation, Beverly, MA). The Qubit DNA HS assay kit (Life Technologies, Carlsbad, CA) was used for DNA quantification. The Ion AmpliSeq Cancer Panel was used to build an amplicon library from 10 ng of DNA from each sample (Life Technologies). Sequence alignment and analysis were performed using Torrent Suite Software (Life Technologies) and lab-developed software (OncoSeek).

## 3. Results

### 3.1. Clinical and Histopathologic Findings

#### 3.1.1. Case 1

**Figure 2:**
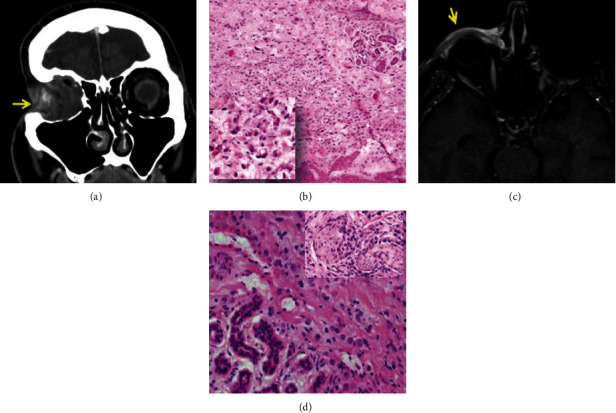
Case 1: (a) CT of maxillofacial area disclosing enhancement along the right infraorbital region (arrow); (b) multifocal cords of signet ring cells infiltrating dermis and directly associated with eccrine dermal ducts (H&E 10x). Inset disclosing single dyshesive signet ring cells infiltrating connective tissue (H&E 20x). Case 2: (c) MRI of the orbit, face, and neck depicting a right anterior mass adjacent to the nasolacrimal duct (arrow); (d) neoplastic cells infiltrating connective tissue and disclosing perineural invasion (inset: 20x).

An 82-year-old male consulted MDACC with a right infraorbital ulcerated subcutaneous nodule and a right nontender neck mass. He had history of SRCC arising in the right orbit diagnosed 13 years prior to presentation at MDACC, followed by radiation therapy, chemotherapy, and right orbital exenteration. His family history revealed lung cancer in one brother, and one sister died of stomach cancer. A CT scan of the maxillofacial area and neck confirmed soft tissue thickening along the right infraorbital region (Figure 2(a)) and metastases to the right intraparotid and right level II lymph nodes. A CT scan of the chest, abdomen, and pelvis showed no evidence of possible second primary disease or metastases. Revision of the right orbital exenteration, parotidectomy, and right neck dissection were performed, and positive resection margins were identified by intraoperative frozen section analysis. The indurated mass involving periorbital tissue and right upper cheek measured 5.3 × 4.5 cm. Microscopically, multifocal cords of signet ring cells arising in close proximity to eccrine dermal ducts were observed (Figure 2(b)), extending into soft tissue and surgical margins of resection. The patient underwent chemoradiation and passed away 5 years after the final treatment (18 years from initial presentation). The case was previously reported by Requena et al. [2].

#### 3.1.2. Case 2

A 65-year-old male with a right lower eyelid mass had history of adenocarcinoma of the left orbit, status post radiation therapy, followed by left orbital exenteration, 12 years prior to presentation at MDACC. An MRI of the orbit, face, and neck performed at MDACC demonstrated a thickened enhancing lesion adjacent to the right nasolacrimal duct measuring 4.1 × 1.0 cm (Figure 2(c)) which extends from the medial canthus to the lateral inferior periorbital soft tissue. In addition, mildly thickened soft tissue enhancement along the left premolar soft tissues suspicious for recurrent disease was reported. Prior positron emission tomography (PET)/CT scan and MRI of the brain performed in an outside facility failed to demonstrate second primary or metastatic disease. Wide local excision of the right lower eyelid and cheek mass was performed with intraoperative margin assessment showing positive margins. Microscopically, cords of histiocytoid cells arising from eccrine glands were identified infiltrating skeletal muscle and orbit with extensive perineural invasion (Figure 2(d)). Surgical margins were positive. Five months after completion of radiation therapy, the patient noted a new right upper eyelid nodule. A CT scan of head and neck reveals local right retroorbital recurrence and left neck adenopathy. A more radical surgery for the right orbit was avoided due to the cancer demonstrating a very slow growth pattern, and it would render him totally blind.

Four years of documented follow-up (until January 2019, when the patient was last contacted at MDACC) after the first surgery done for the lower eyelid mass, he was still alive. Currently, the patient is being seen at an outside facility with his only eye globe intact. He has had slowly progressive distant lymph node metastatic disease for which standard treatments have not been offered, and he is not keen to enroll in clinical trials.

#### 3.1.3. Case 3

**Figure 3:**
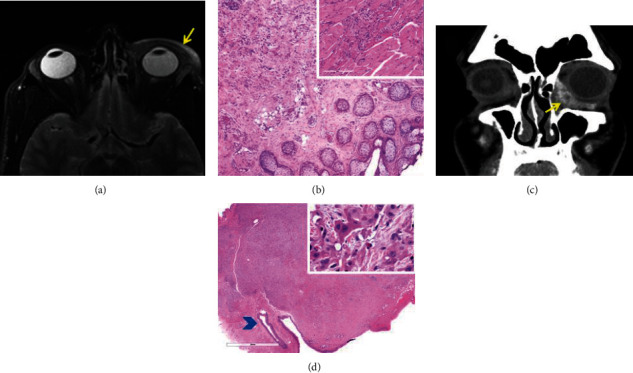
Case 3: (a) MRI depicting an infiltrative lesion involving pre- and postseptal regions of the left upper and lower eyelids (arrow); (b) tarsus diffusely infiltrated by neoplastic cells (H&E 4x). Inset: higher magnification of cords of neoplastic cells infiltrating skeletal muscle (20x). Case 4: (c) CT scan shows enhancing and infiltrative mass along the inferomedial aspect of the left anterior orbit; (d) panoramic view shows neoplastic cells forming a mass lesion adjacent to the punctum (arrowhead) and infiltrating caruncle (H&E). Inset: higher magnification depicting tumor cells with vesicular nuclei, large nucleoli, and eosinophilic cytoplasm with intracytoplasmic vacuolization (H&E 20x).

A 51-year-old male presented to MDACC for evaluation of a left upper eyelid mass. On review of the outside needle core biopsy sections, scant histiocytoid cells (CK7 positive) were noted percolating among normal lacrimal gland tissues. The microscopic assessment was a poorly differentiated adenocarcinoma with indeterminate origin due to the limited biopsy sample. An MRI of orbit and face showed an infiltrative lesion involving pre- and postseptal regions of the left upper and lower eyelids, the lacrimal gland, and the focal intraconal extension (Figure 3(a)). The patient's previous PET/CT scan was negative for a second malignancy. Exenteration of the left orbit with intraoperative evaluation of the margins was performed. Macroscopically, the tumor measured 5.5 × 2.5 cm. Histopathologically, clusters of atypical histiocytoid cells involving the dermis, tarsus, palpebral conjunctival, and periorbital soft tissues were present (Figure 3(b)). Despite wide excision beyond the clinically visible mass, tumor cells were seen along the cauterized resection margins. Postoperative adjuvant chemoradiation therapy was completed, and at 12-month follow-up, there was no evidence of recurrence.

#### 3.1.4. Case 4

A 40-year-old male consulted MDACC with a history of thickening of the left medial lower eyelid and enlargement of a caruncle. A CT scan of orbits and head and neck soft tissue demonstrated an enhancing and infiltrative mass along the inferomedial aspect of the left anterior orbit (Figure 3(c)) and no evidence of facial or cervical lymphadenopathy. PET/CT scan performed in an outside facility did not detect second primary malignancy or metastatic disease. Radical surgical resection of the left orbital, eyelid, and lacrimal sac mass with intraoperative assessment of margins was performed. Grossly, the specimen measured 4.0 × 3.5 cm. Histopathologically, neoplastic cells forming a mass lesion adjacent to the punctum and infiltrating caruncle was seen (Figure 3(d)). Cords of pleomorphic cells with histiocytoid appearance infiltrating connective tissue and depicting vesicular nuclei, large nucleoli, and eosinophilic cytoplasm with intracytoplasmic vacuolization were identified (Figure 3(d) inset). Perineural invasion was present. The patient underwent proton therapy and chemotherapy. Thirteen months after treatment, the patient had a left neck dissection with metastatic adenocarcinoma to two of six lymph nodes at level III.

### 3.2. Summary of Immunohistochemical Findings

Immunohistochemical studies are summarized in Table 2.

**Figure 4:**
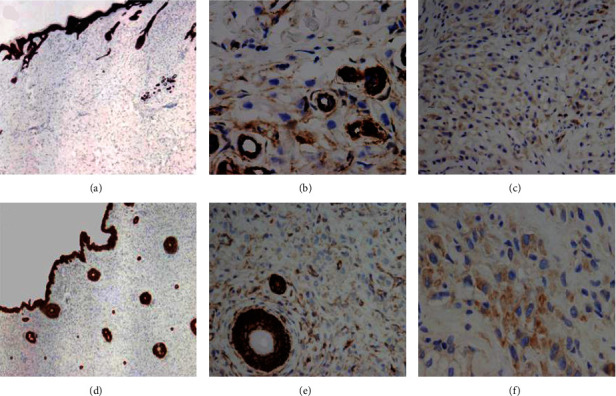
Case 1: (a) neoplastic cells show loss of E-cadherin expression (4x); (b) weak heterogeneous membranous staining for *β*-catenin is seen focally (20x); (c) low cytoplasmic expression of Src (10x). Case 2: (d) tumor cells depicting loss of E-cadherin expression (4x); (e) heterogeneous, incomplete membranous staining for *β*-catenin is present (10x); (f) focal low cytoplasmic staining for Src in less than 25% of tumor cells (20x).

**Figure 5:**
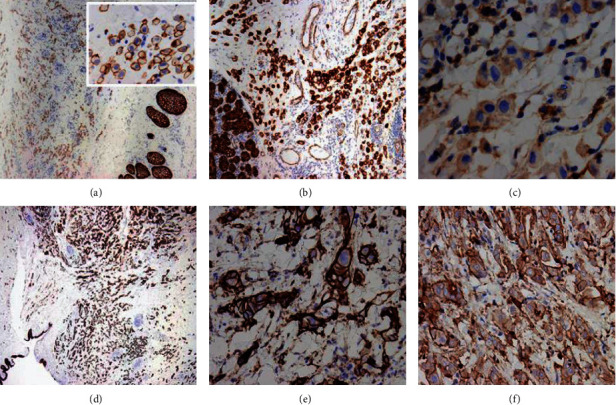
Case 3: (a) tumor cells and normal sebaceous glands retain positive staining for E-cadherin (inset: depicting complete membranous staining; 20x); (b) tumor cells and lacrimal gland acini show uniform, membranous *β*-catenin staining (10x); (c) high cytoplasmic/membranous staining for Src is identified in >50% of tumor cells (20x). Case 4: (d) strong E-cadherin expression is present (4x); (e) strong membranous and cytoplasmic staining for *β*-catenin (20x); (f) increased cytoplasmic expression of Src is noted in >50% of tumor cells (20x).

Immunohistochemically, the tumor cells showed strong-diffuse expression of CK7 which also highlighted the distribution of cells within the resected tissues, notably sparing the epithelium, and demonstrating predominantly single-cell growth with some small clusters and rare duct formation. In cases 1 and 2, the neoplastic cells showed complete loss of E-cadherin expression within single and clusters of tumor cells with heterogeneous membranous staining for *β*-catenin and low cytoplasmic expression of Src (Figure 4). In contrast, the other two cases (cases 3 and 4) showed intact, strong diffuse membranous E-cadherin expression along with homogeneous, membranous *β*-catenin expression and increased cytoplasmic Src expression (Figure 5).

Cyclin-D1 was positive in three of our cases, two of them demonstrating preserved E-cadherin/*β*-catenin expression. Strong immunoreactivity for p53 (>50% of tumor cells) was observed as well as focal positive nuclear staining for c-Myc in case 4. Additional immunohistochemical studies were performed in three of our cases (cases 2, 3, and 4) which were received for consultation from outside facilities. The three cases were positive for gross cystic disease fluid protein 15 (GCDFP-15) stain while negative for Her2Neu. Androgen receptor (AR) was positive in cases 2 and 4.

### 3.3. Molecular Diagnostics-Solid Tumor Genomics Assay v1

#### 3.3.1. Case 1

No mutations or copy number changes were identified. Germline polymorphisms were noted on *TSC2* (missense mutation, c.4136C>T p.S1379L) and *FGFR4* (missense mutation, c.743G>A p.R248Q). The polymorphism phenotyping detected on *FGFR4* was probably deleterious.

#### 3.3.2. Case 2

Somatic mutations were detected on *PIK3CA* (E545K and N1044K) and one *CDH1* mutation was found in exon 13 (frameshift). Copy number variations were not identified (Table 3).

#### 3.3.3. Case 3

No mutation or copy number changes were detected. Germline polymorphisms were noted on *PTEN* (missense mutation, c.235G>A p.A79T) and *MLH1* (missense mutation, c.1217G>A p.S406N). The polymorphism phenotyping was considered benign in both genes.

#### 3.3.4. Case 4

Somatic mutations were detected in *PIK3CA* (H1047R) and *TP53* (D259Y). Benign germline polymorphism was noted on *ERBB2* (missense mutation, c.3115G>A p.A1039T). No copy number changes were noted (Table 3).

## 4. Discussion

Clinical presentation of primary SRC/Histiocytoid carcinoma of the eyelids is usually nonspecific, with main complaint of gradual swelling or thickening of eyelid which might then extend to the other eyelid of the same eye, giving the typical “monocle-like” appearance, or involves the contralateral eyelids [1]. The involvement of the contralateral eye and the absence of metastasis suggest local spread from the initial lesion; however, the possibility of a second primary tumor or metastasis cannot be excluded. This clinical presentation may mimic inflammatory processes such as blepharoconjunctivitis, chalazion, orbital cellulitis, eyelid ptosis, and cutaneous metastasis from other organs. Frequently, tumor involvement extends beyond the lesion that may be clinically observed, requiring more extensive surgery and not uncommonly encountering positive surgical margins. Local spread may occur by lymphatic channels [4]. Surgical resection with strict histologic evaluation of the resection margins is essential for local-regional control.

An appropriate metastatic workup to exclude other primary sites of adenocarcinoma must be considered. The tumor cells usually spare the epidermis but infiltrate diffusely adjacent structures including tarsus, skeletal muscle, palpebral conjunctiva, and soft tissue of the orbit with extensive perineural invasion. A mild to moderate inflammatory infiltrate is also present in this tumor.

Three variants of primary eccrine adenocarcinoma of the eyelid have been described: ductal, mucinous, and adenoid cystic. Well-differentiated ductal carcinoma with signet ring cells has been classified as signet ring cell carcinoma, while poorly differentiated ductal carcinoma is classified as the histiocytoid variant [5].

Most tumor cells in our series predominantly resemble histiocytes with eosinophilic, abundant cytoplasm, some of them with intracytoplasmic vacuoles and signet ring cells arising in close proximity to eccrine dermal ducts, features consistent with the histiocytoid variant of ductal adenocarcinoma of the eccrine sweat glands.

On the other hand, mucinous sweat gland adenocarcinoma is microscopically characterized by lobules of tumor cells floating in large pools of mucin. Clinically, mucinous sweat gland adenocarcinoma of the eyelid usually demonstrates a period of no growth for several years followed by a rapid growth, associated with pain. It has an indolent course with local recurrence following excision, but the rate of metastasis is low (9.6%) [6–9, 22]. Primary cutaneous adenoid cystic carcinoma is the rarest of eccrine sweat gland carcinomas of the eyelid. It is most commonly observed in the skin of the scalp and chest, arising from the palpebral portion of the lacrimal gland and accessory lacrimal glands of the conjunctiva. It can be differentiated from the adenoid variant of basal cell carcinoma of the eyelid by lack of continuity with the epidermis or hair shafts and the absence of peripheral palisading of the nuclei [23].

Review of the literature indicates that primary SRC/Histiocytoid carcinoma of the eyelid shows strong and diffuse reactivity for CK7, CAM 5.2, high molecular weight cytokeratin (HMWCK), keratin cocktail AE1/AE3 and MNF116, carcinoembryonic antigen (CEA), epithelial membrane antigen (EMA), gross cystic disease fluid protein-15 (GCDFP-15), p63, mucin-1 (MUC-1), and BerEP4; moderate positivity for (alpha)-smooth muscle actin (ASMA), tissue-specific transcription factor 1, MUC-2, podoplanin, N-cadherin, and E-cadherin; and weak positivity for epidermal growth factor receptor (EGFR) [2].

Immunoreactivity for E-cadherin, in SRC/Histiocytoid carcinoma of the eyelid, has been studied in prior case reports [1–3, 7]; however, the evaluation of E-cadherin/*β*-catenin complex expression has not been described previously. Underexpression of the E-cadherin protein complex is found in gastric and other cancers such as colon, lobular carcinoma of the breast, lung, prostate, and in plasmacytoid-variant bladder carcinoma [12–16, 24–26].

It has been proposed that the loss of E-cadherin-mediated cell adhesion is a prerequisite for tumor cell invasion and metastasis formation [12, 27]. E-cadherin interacts, intracellularly, with catenins forming a complex that maintains normal epithelial polarity and intercellular adhesion and regulates cellular differentiation and proliferation. *β*-Catenin is a multifunctional cytoplasmic protein and has been found to be an important member in the Wnt signaling pathway, which plays important roles in cellular development, proliferation, and differentiation. Conventionally, Wnt signaling causes *β*-catenin accumulation in a complex with T cell factor/lymphoid enhancer factor (TCF/LEF), which regulates target gene expression. Reduction and loss of *β*-catenin or other molecules might disrupt stability and integrity of the E-cadherin-catenin complex and disturb cellular adhesive junction. Additionally, in the absence of Wnt signals, *β*-catenin is sequestered in a complex with the adenomatous polyposis coli tumor suppressor (*APC*), *AXIN*, and a serine threonine glycogen synthetase kinase-3*β* (GSK-3*β*), enabling phosphorylation and degradation of free *β*-catenin by the ubiquitin-proteasome system, processes that have been associated with a wide variety of human malignancies [28]. Nelson and Nusse [29] have described that abnormal expression of E-cadherin and *α*,*β*-catenins significantly correlated with differentiation, and lymph node and liver metastases of pancreatic cancer.

The structural and functional integrity of the cadherin-catenin complex is regulated by phosphorylation. Serine/threonine phosphorylation of *β*-catenin or E-cadherin results in increased stabilization of the cadherin-catenin complex while phosphorylation by nonreceptor tyrosine kinases such as *Src* or epidermal growth factor receptor (EGFR) disrupts binding of *β*-catenin to cadherin [29]. *Src* is an integral part of a cadherin-activated cell signaling pathway that can positively regulate adhesion and cell contact integrity as mentioned by McLachlan et al. [28]. However, *Src* has a bimodal impact on cadherin function, exerting a positive supportive role at lower signal strengths, but inhibiting function at high signal strengths which suggests that quantitative changes in signal strength can lead to distinctly different functional outcomes. One potential pathway for *Src* to influence cadherin function is through PI3-kinase which is capable of supporting cell-cell adhesion when it is active at cadherin cell-cell contacts. Moreover, *Src* might participate as an upstream element in signaling pathways that activate PI3-kinase [28].

*Cyclin D1* and *c-Myc* genes are important cell cycle regulators playing important roles in the control of cell growth, differentiation, apoptosis, and neoplastic transformation. The correlation between abnormal expression of *β*-catenin and overexpression of target genes, *Cyclin D1* and *c-Myc*, in pancreatic cancer and colorectal carcinoma have been previously evaluated and correlated with loss of tumor differentiation, metastasis and prognosis [18, 30]. The activation of these target genes has also been associated with inactivating mutations of the *APC* gene as well as alteration in the Wnt–*β*-catenin signaling pathway.

Destabilization of epithelial adherens junctions is present during EMT. E-cadherin is cleaved at the plasma membrane and subsequently degraded. *β*-Catenin can no longer interact with E-cadherin, and it is either degraded or protected from degradation [27]. EMT progresses, and the expression of junction proteins is transcriptionally repressed which stabilizes the loss of epithelial junctions.

Herein, we present four male patients with histopathologic diagnosis of SRC/Histiocytoid carcinoma of the eyelid arising in the eccrine glands with ages between 40 and 69 years at the initial diagnosis. Metastatic tumors from the breast, gastrointestinal tract, and urothelial lining have been excluded. Although, histopathologically, the tumors (cases 1, 2, and 3) disclose predominantly an infiltrative, diffuse growth pattern of histiocytoid cells, more cohesive neoplastic cells with signet ring cells are noted in case 4.

An association between the absence/abnormal expression of E-cadherin/*β*-catenin and the decreased cytoplasmic expression of Src in cases 1 and 2 is observed while an intact/strong expression of E-cadherin/*β*-catenin is seen along with a high cytoplasmic expression of Src in cases 3 and 4.

Karayiannakis et al.[18] describe an abnormal expression of *β*-catenin with a heterogeneous pattern of staining in ductal carcinoma in situ of the breast (54%) with high concordance between *β*-catenin expression patterns in *in situ* and invasive components (81%). We observe a similar heterogeneous pattern of staining in cases 1 and 2, which might indicate that changes in *β*-catenin expression may occur early during the development of these tumors. The retained expression of E-cadherin/*β*-catenin with positive/high cytoplasmic expression for Src, seen in cases 3 and 4, could be associated with an abnormality in different stages of the convergence of the *β*-catenin and cadherin pathways which could also be supported by the positive expression of p53, c-Myc, and Cyclin-D1 as noted in case 4.

Although there is an abnormal expression of *β*-catenin (case 1) associated with positive Cyclin-D1 expression, cases 3 and 4 demonstrate normal *β*-catenin expression with also positive Cyclin*-*D1 expression. Guo et al. [31] indicate that an association exists among the abnormal expression of *β*-catenin and positive expression of Cyclin-D1 in patients with breast cancer, and they also report that breast cancer patients with normal expression of *β*-catenin and positive expression of Cyclin-D1 exhibit longer tumor-free survival time.

The *CDH1* gene provides instruction for making E-cadherin protein and is the best known gene associated with the hereditary diffuse gastric cancer syndrome (HDGC). The literature review demonstrates that mutation in the *CDH1* gene predisposes primarily to diffuse gastric cancer (DGC). Multiple DGC cases in a family, DGC at a young age in an individual, or the combination of DGC and lobular breast cancer (LBC) in an individual or a family defines the hereditary DGC syndrome. Testing for germline *CDH1* mutations is warranted in HDGC [32]. Approximately 40% of HDGC families have germline mutations in *CDH1* [15]. However, Lee et al. [33] report that 35.7% of their DGC samples showed *CDH1* somatic mutations and the frequencies of *CDH1* somatic mutations, previously reported, can vary from 3% to greater than 50%. In the same work, *PIK3CA* and *TP53* are found the most frequently mutated genes in DGC and intestinal gastric cancer. *PIK3CA* is an oncogene whose mutated form exhibits increased kinase activity, causing cancer cell proliferation and the relative high mutation of this gene in DGC may reflect the specificity of mutations in this gene to DGC.

The somatic mutations on the *CDH1* (case 2), *PIK3CA* (cases 2 and 4), and *TP53* genes, detected in the present study, indicate abnormalities in the E-cadherin structure as well as in the calcium signaling pathway which is essential for the function of E-cadherin as has been previously reported by Lee et al. [33]. We can also suggest that the high cytoplasmic expression of Src observed in case 4 demonstrates an alteration in the PI3-kinase pathway which also participates in the cadherin functions [28].

*PIK3CA* mutation in the hotspot E545K is detected in case 2 which is a well-known mutation in DGC and other malignancies [33]. In addition, mutations in *CDKN1B* and *NTRK3* genes have been recently found associated with this tumor [3]. These genes also play a role in the function of E-cadherin as well as in the mechanisms related to the epithelial-mesenchymal transition.

Although our molecular studies reveal no somatic mutations in cases 1 and 3, further research studies are mandatory to determine and understand the different interconnecting molecular pathways participating in the pathogenesis of this tumor. Up to date, there are just few investigations targeting cancer-related genes associated with this malignant neoplasm. We believe that this eyelid tumor is biologically similar to primary SRC/Histiocytoid carcinoma described in the stomach and breast, among others with alterations in expression of the E-cadherin/*β*-catenin complex and associated molecular pathways. The spectrum of histological, immunohistochemical, and molecular findings in this malignancy might represent abnormalities in the different stages of the convergence of E-cadherin/*β*-catenin pathways which affect the clinical outcomes seen in these patients. All four patients received adjuvant chemotherapy and/or radiotherapy with three developing recurrent disease. One of those patients (case 1) died five years after his final treatment (18 years after initial presentation).

While E-cadherin abnormalities cannot be specifically targeted, clinical research trials aiming at associated pathways are being evaluated for therapeutic consideration in similar tumors in other sites. The molecular heterogeneity identified in this study emphasizes the need to consider genetic testing in patients with SRC/Histiocytoid carcinoma of the eyelid to gain further insight into this rare disease. Moreover, with improved understanding of altered biologic pathways, patients may ultimately benefit from targeted personalized therapy.

## Figures and Tables

**Table 1:** Fifty cancer-related genes utilizing Ion AmpliSeq™ Cancer HotSpot v2 Panel.

Gene				
ABL1	EGFR	GNAQ	KRAS	PTPN11
AKT1	ERBB2	GNAS	MET	RB1
ALK	ERBB4	HNF1A	MLH1	RET
APC	EZH2	HRAS	MPL	SMAD4
ATM	FBXW7	IDH1	NOTCH1	SMARCB1
BRAF	FGFR1	IDH2	NPM1	SMO
CDH1	FGFR2	JAK2	NRAS	SRC
CDKN2A	FGFR3	JAK3	PDGFRA	STK11
CSF1R	FLT3	KDR	PIK3CA	TP53
CTNNB1	GNA11	KIT	PTEN	VHL

**Table 2:** Immunohistochemical results for four SRC/Histiocytoid carcinomas of the eyelid.

Antibody	Case 1	Case 2	Case 3	Case 4
CK7	Positive	Positive	Positive	Positive
CK20	Negative	Negative	Negative	Negative
p63	Negative	Negative	Negative	Negative
p53	Negative	Negative	Negative	Positive^+++^
E-cad	Negative^a^	Negative^a^	Positive	Positive
*β*-Catenin	Abnormal^b^	Abnormal^b^	Positive	Positive
Src	Low^c^	Low^c^	High^d^	High^d^
c-Myc	Negative	Negative	Negative	Positive^+^
Cyclin-D1	Positive^++^	Negative	Positive^+^	Positive^+^
GCDFP-15	Not performed	Positive^f^	Positve^f^	Positive^f^
AR	Not performed	Positive	Not performed	Positive
Her2Neu	Not performed	Score 2+^e^	Score 1+	Score 1+

^a^Negative: <10% of the tumor cells staining for E-cadherin. ^b^Abnormal: heterogeneous, incomplete membranous staining for B-catenin. ^c^Decreased/low Src cytoplasmic staining in less than 25% of tumor cells. ^d^High cytoplasmic Src expression in 25%-75%of tumor cells. ^e^Her2 FISH was negative. ^f^Performed in an outside facility. ^+^Focal positivity (1%-<25% of tumor cells). ^++^Moderate positive expression in >25%-<50% of tumor cells. ^+++^Strong positive immunoreactivity seen in >50% of tumor cells.

**Table 3:** Summary of somatic mutations of cases 2 and 4.

Gene	Standardized Nomenclature (HGVS)	Location	DNA change	Protein change	dbSNP ID	Cosmic ID
Case 2						
*PIK3CA*	NM_006218.2(PIK3CA):c.1633G>Ap.E545K (VAF: 13.6%)	Exon 10	SNV	Missense	rs104886003COSM763	
*PIK3CA*	NM_006218.2(PIK3CA):c.3132T>Ap.N1044K (VAF: 14.7%)	Exon 21	SNV	Missense		COSM12592
*CDH1*	NM_004360.3(CHD1):C.1991_1992InsTAp.K664fs (VAF: 28.8%)	Exon 13	Insertion	Frameshift		

Case 4						
*PIK3CA*	NM_006218.2(PIK3CA):c.3140A>G p.H1047R (MAF: 14%)	Exon 21	SNV	Missense	rs121913279	COSM775
*TP53*	NM_000546.5(TP53):c.775G>T p.D259Y (MAF: 16%)	Exon 7	SNV	Missense		COSM11552

## Data Availability

The supporting data of this study are available on request from the corresponding author (Maria Del Valle Estopinal, MD).
